# Evaluation of Fungistatic Activity of Eight Selected Essential Oils on Four Heterogeneous *Fusarium* Isolates Obtained from Cereal Grains in Southern Poland

**DOI:** 10.3390/molecules25020292

**Published:** 2020-01-10

**Authors:** Teresa Krzyśko-Łupicka, Sławomir Sokół, Anna Piekarska-Stachowiak

**Affiliations:** 1 Institute of Environmental Engineering and Biotechnology, Faculty of Natural and Technical Sciences, University of Opole, Kominka 6A, 45-035 Opole, Poland; 2 Institute of Biology, Faculty of Natural and Technical Sciences, University of Opole, Oleska 22, 45-052 Opole, Poland; sokol@uni.opole.pl; 3 Institute of Biology, Biotechnology an Environmental Protection, Faculty of Natural Sciences, University of Silesia in Katowice, Jagiellońska 28, 40-032 Katowice, Poland; anna.piekarska@us.edu.pl

**Keywords:** fungistatic activity, *Fusarium*, *F. avenaceum*, *F. culmorum*, *F. graminearum*, *F. oxysporum*, thymol, citral

## Abstract

The aim of the study was to determine the relationship between the chemical composition of eight commercial essential oils (EsO) (garlic, grapefruit, lemon grass, tea tree, thyme, verbena, cajeput, and *Litsea cubeba*) and their fungistatic activity in relation to four species of *Fusarium*: *F. avenaceum*, *F. culmorum*, *F. graminearum*, and *F. oxysporum*. The species identification of *Fusarium* isolates was confirmed by matrix-assisted laser desorption/ionization time-of-flight (MALDI-TOF) mass spectrometer. The determination of qualitative and quantitative chemical composition of the EsO was carried out using the gas chromatography–mass spectrometry (GC–MS) method. The fungistatic activity of EsO was assessed by using the method of poisoned substrates. The data were compiled in the STATISTICA 13.0 program. The chemical composition of the tested oils varied; the dominant fraction, except for grapefruit and garlic oils, were monoterpenoids. The greatest similarity to the action of the synthetic pesticide Funaben T was found in four oils, i.e., thyme, lemongrass, verbena, and *Litsea cubeba*. The studies showed that *F. oxysporum* and *F. avenaceum* were characterized by a higher resistance to low oil concentrations, and *F. culmorum* and *F. graminearum* by sensitivity. The fungicidal activity of two EsO-dominant monoterpenoids-thymol and citral—has been confirmed.

## 1. Introduction

Essential oils (EsO) of oil-giving vascular plants are compositions of various chemical compounds, including, among others, secondary metabolites. In EsO, terpenes and their derivatives, such as citral, eugenol, eucalyptol, germacrene, carvacrol, limonene, and thymol are present as dominant components. The content of individual chemical compounds in a given oil is variable and depends on many factors, such as genetic conditions, vegetation advancement, geographical origin, time of harvesting and storage of plants, as well as the technique of obtaining and storing EsO [1,2,3]. Orłowska [4] showed differences in the composition of thyme oils extracted from 18 species belonging to the genus of thyme (*Thymus* L.); she identified only one common volatile compound, β-linalool in these oils, and only 10 of the tested species in the composition of the volatile fraction contained a mixture of isomers, such as thymol and/or carvacrol, in quantities of 38–42%.

The unique chemical composition is shown by oils obtained from garlic (*Alium sativum*) because they contain dominant organosulphur compounds as components. Their content is diversified, which is indicated, among others, by Kędzia [5]. In this paper, it was shown that the chemical composition of garlic oils from Mexico, France, Egypt, Turkey, and China is diverse, with the following compounds: allyl methyl and diallyl sulphides; allyl methyl and alkyl disulphides; dimethyl, allyl methyl, and diallyl trisulphides. Individual types of oils may therefore differ significantly both in their qualitative and quantitative composition and in the proportions between them.

The substances contained in the EsO of vascular plants exhibit interactions of a biochemical nature (both harmful and beneficial), in systems: plants-plants, microorganisms-microorganisms, and plants-microorganisms. In 1937, Hans Molish introduced the concept of allelopathy [6,7]. EsO may exhibit fungistatic activity characterized by partial inhibition of mycelial growth or fungicidal, causing complete inhibition of the development of a given species, comparable to those of synthetic fungicides [8]. The effect of the action depends on both the sensitivity of the fungus and the chemical composition and concentration of EsO.

The range of EsO biocidal properties was tested in numerous studies. It was shown that the differentiation of individual components contained in EsO affects their properties and bioactivity. According to Cavanagh and Wilkinson [2], Bakkali et al. [3], and Sienkiewicz et al. [1], the effectiveness of oils is determined by the chemical composition (qualitative and quantitative) of the active substance contained in the oil. High biological activity compounds include the following: terpinene, limonene, germacrene, citronellyl acetate, caryophyllene, thymol, carvacrol, eugenol, eucalyptol, terpineol, and linalool [8,9,10,11].

Numerous studies showed that EsO exhibit antifungal properties, manifested by inhibition or restriction of mycelium development, inter alia, *Fusarium* [11,12,13,14]. Fungal growth disorders are caused by changes in the structure of fungi associated with the interaction of EsO on the enzymes responsible for cell wall synthesis. Inhibition of fungal growth by oils can be synonymous with changes in their ultrastructure. An important role in the degradation of the fungal cell membrane is attributed to lyophilic and polar compounds contained in oils. Lyophilic compounds disturb the structure of the cell membrane, inhibit its synthesis, form spores, and impede the respiration process. In contrast, polar compounds with active chemical groups participate in the degradation of the cell membrane. In sensitive fungi species, there are changes in the structure of the cell membrane and in the composition of fatty acids and the formation of vacuoles in the cells of fungi. In parallel, the synthesis and regeneration of cell membrane components is inhibited, which inhibits cell growth and division as well as spore production [15,16].

By selecting EsO properly, we can achieve a significant fungicidal effect even when using oils in very low concentrations. Thus, some oils can be used in practice, in biological protection of plants against *Fusarium* phytopathogens [13]. Currently, *Fusarium* polyphagous fungi are controlled in crops with the use of chemical fungicides, in which active substances belonging to various chemical groups dominate (triazoles, imidazoles, morpholines, oxazolidines, and benzimidazoles). Sometimes, mixtures of these substances are also used. However, in recent years, despite the proper selection of fungicides, an increasing resistance of *Fusarium* fungi was observed, which is a frequent phenomenon in the common use of limited arsenal of agents [17].

The aim of this study was to determine the relationship between the chemical composition of eight commercial EsO and their fungistatic activity in relation to four isolates from genus *Fusarium* (*F. avenaceum*, *F. culmorum*, *F. graminearum*, and *F. oxysporum*).

The present experiment was established in such a way that it was possible to identify variable spectrum of compounds and groups of compounds present in different EsO to estimate fungistatic activity. EsO and/or ingredients of EsO could potentially be useful in the formation of alternative fungicides. Synthetic fungicides are relatively high ecotoxic and resulting fungal resistance reactions.

## 2. Results

### 2.1. Results of Determination of Isolates of Individual Fusarium sp.

**Figure 1:**
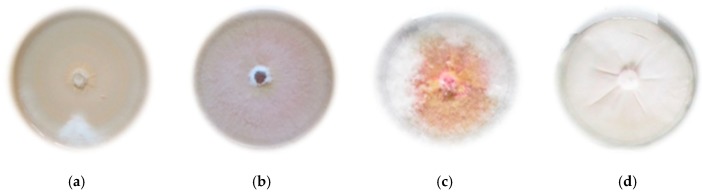
Morphological features of the tested species of the Genus *Fusarium* on PDA medium (Potato Dextrose Agar): (**a**) *F. avenaceum*; (**b**) *F. culmorum*; (**c**) *F. graminearum*; (**d**) *F. oxysporum*.

From 19 *Fusarium* isolates of wheat grains, one representative for each of the four species was selected. The isolate with the highest identification value (matrix-assisted laser desorption/ionization time-of-flight (MALDI-TOF) mass spectrometer) was selected (Table 1), showing typical features of cultures of a given species (Figure 1).

### 2.2. Results of the Determination of the Qualitative and Quantitative Composition of Essential Oils

Chemical analysis of EsO showed the presence of about a hundred different compounds with the content from a per mil to several dozen percent, belonging to different chemical groups. Table 2 shows data on 7 EsO except for garlic, whose chemical composition was different from the analyzed oils. Garlic oil contained only organosulphur compounds, such as diallyl trisulphide (46.31 ± 0.37%), diallyl disulphide (22.62 ± 0.24%), allyl methyl trisulphide (21.46 ± 0.29%), diallyl monosulphide (5.22 ± 0.11%), allyl methyl disulphide (3.34 ± 0.07%), and dimethyl trisulphide (1.05 ± 0.13%).

The tested oils contain a small number of compounds whose concentration in the oil exceeded 50%. These were the groups of organosulphur compounds (100%) in garlic oil, citral, lemongrass oils (68.94%), and *Litsea cubeba* (61.72%). In a slightly lower concentration (45.74%), thymol was found in thyme oil.

In concentrations higher than 25% and lower than 50%, four compounds were present: 1-terpinen-4-ol (38.24%) in tea tree oil; α-terpineol (36.57%) in cajeput oil; citral (36.00%) in verbena oil; and limonene (34.63%) in grapefruit oil. On the other hand, five compounds were present in concentrations ranging from 10% to 25%, i.e., limonene in *Litsea cubeba* (20.94%) and thyme oils (15.15%); eucalyptol in cajeput (18.50%), tea tree (13.90%) and verbena oils (13.45%); α-terpineol in verbena (18.26%) and lemongrass oils (10.29%); 3-caren (17.04%) in tea tree; and linalool (11.19%) in cajeput oil (Table 2).

The chemical composition of the individual studied EsO varied both in terms of quality and quantity. Individual compounds belonged to different chemical groups. Garlic oil contained only organosulphur compounds (100%).

Other oils (thyme, lemongrass, verbena, and cajeput) were characterized by a high content of monoterpenoids (59.02–87.17%). However, in the composition of lemongrass oil and *Litsea cubeba*, aliphatic monoterpenoids prevailed (76.35% and 66.17%, respectively), and in thyme, tea tree and cajeput oils, monocyclic monoterpenoids were present in the largest quantities, i.e., from 41.85% to 48.08%.

The grapefruit oil was dominated by monoterpenes (45.64%), mainly monocyclic (35.49%), and a high content of compounds not belonging to terpenes, which constituted approximately 32% of the total composition of this oil.

In verbena oil, aliphatic monoterpenoids (44.53%), monocyclic monoterpenoids (23.24%) and bi- and tricyclic monoterpenoids (19.4%) were found in high concentrations. It should also be emphasized that in thyme and *Litsea cubeba* oils, characterized by high monoterpenoid content, aliphatic monoterpenes were present in large quantities (26.44% and 20.94%, respectively) (Table 3 and Table 4).

To sum up, the chemical composition of oils taking into account the chemical groups of compounds and the proportions between them, as well as the dominant and additional components, is presented below (Table 2, Table 3 and Table 4):

**Garlic oil (C)**—only organosulphur compounds (100%) were present, such as diallyl trisulphide (46.31 ± 0.37%), diallyl disulphide (22.62 ± 0.24%), allyl methyl trisulphide (21.46 ± 0.29%), diallyl monosulphide (5.22 ± 0.11%), allyl methyl disulphide (3.34 ± 0.07%) and dimethyl trisulphide (1.05 ± 0.13%).
**Lemongrass oil (L)**—aliphatic monoterpenoid-citral (68.94%) was found to be a dominant constituent; other compounds were present in concentrations significantly lower than 10%, so it was established that the additional compound can be aliphatic monoterpenoid-linalool (5.73%). The ratio of monoterpenoids to monoterpenes was approximately 17:1.
***Litsea cubeba* oil (LC)**—aliphatic monoterpenoid-citral (61.72%) was the dominant constituent; however, an additional constituent was monocyclic monoterpene-limonene (20.94%). Other constituents occurred in low concentrations, less than 5%. The ratio of monoterpenoids to monoterpenes was approximately 2:1.
**Thyme oil (T)**—the dominant constituent was monocyclic monoterpenoid-thymol (45.75%); an additional constituent was monocyclic monoterpene-limonene (15.15%). Other compounds were present in concentrations lower than 10%. The presence of aliphatic monoterpenoids-linalool (8.90%) and monocyclic monoterpene-γ terpinene (8.10%) is noteworthy. The ratio of monoterpenoids to monoterpenes was approximately 2:1.
**Tea tree oil (TTO)**—monocyclic monoterpenoid-1-terpinen-4-ol (38.24%) was the dominant constituent; an additional constituent was monoterpene bi- and tricyclic—3-caren (17.04%); other compounds were present in lower concentrations, but exceeding 10%, i.e., monoterpenoid 2-3 cyclic eucalyptol (13.90%) and monocyclic monoterpene α-terpinene (10.29%). The ratio of monoterpenoids to monoterpenes was approximately 2:1.
**Cajeput oil (K)**—monoterpenoids were present as dominant and auxiliary constituents; monocyclic—was the dominant α-terpineol (36.57%), and 2–3 cyclic—eucalyptol (18.50%) was the additional. The presence of the aliphatic monoterpenoid linalool (11.19%) is also worth mentioning. The ratio of monoterpenoids to monoterpenes was approximately 6:1.
**Verbena oil (V)**—the dominant and additional constituents were monoterpenoids; aliphatic—citral (36.0%) (dominant) and monocyclic monoterpenoid-α-terpineol (18.26%) (additional). The presence of bi- and tricyclic monoterpenoids-eucalyptol (13.46%) is also worth mentioning. The ratio of monoterpenoids to monoterpenes was approximately 10:1.
**Grapefruit oil (G)**—the dominant constituent was monocyclic monoterpene-limonene (34.6%), and aliphatic monoterpene-β myrcene (5.32%) or aliphatic monoterpenoid-linalool (4.83%) can be considered as additional constituents. Other numerous constituents were present in small quantities up to 4%. High content of auxiliary heterogeneous substances, not terpenes’ (32.09%) was noted. The ratio of monoterpenoids to monoterpenes was approximately 1:3.

### 2.3. Results of the Assessment of Fungistatic Activity of Essential Oils and Their Influence on Individual Fusarium ssp.

**Figure 2:**
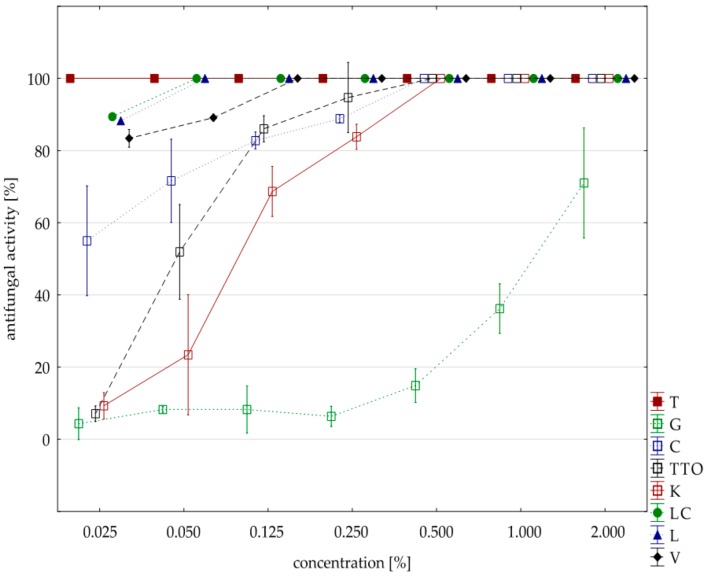
Plot of antifungal activity vs. concentration for *F. culmorum*.

The analysis of fungistatic activity of eight EsO showed differences in sensitivity/resistance of individual isolates to the effect of these oils. The most sensitive isolate was *F. culmorum*. Seven oils, except for the grapefruit, showed 100% fungistatic effect, but it depended on the applied oil concentration. Thyme oil inhibited the growth of this isolate at the lowest concentration (0.025%). Lemongrass oils and *Litsea cubeba* caused the same result at slightly higher concentrations (0.050%), and verbena oil at (0.125%). Garlic, cajeput, and tea tree oils, which were only effective at a concentration of 0.500%, had a weaker effect. Grapefruit oil had the weakest effect and only at a concentration of 2.000% did it show fungistatic activity amounting on average to 71.03% (Table 5, Figure 2). The result of Kruskal–Wallis test (H (9, n = 230) = 125.87, *p* = 0.00) showed that the differences in fungistatic activity of the oils used were statistically significantly. In addition, the Kruskal–Wallis test (H (3, n = 83) = 4.69, *p* = 0.20) showed that there are no statistically significant differences between Funaben T fungistatic activity and thyme, lemongrass, and *Litsea cubeba* oils.

**Figure 3:**
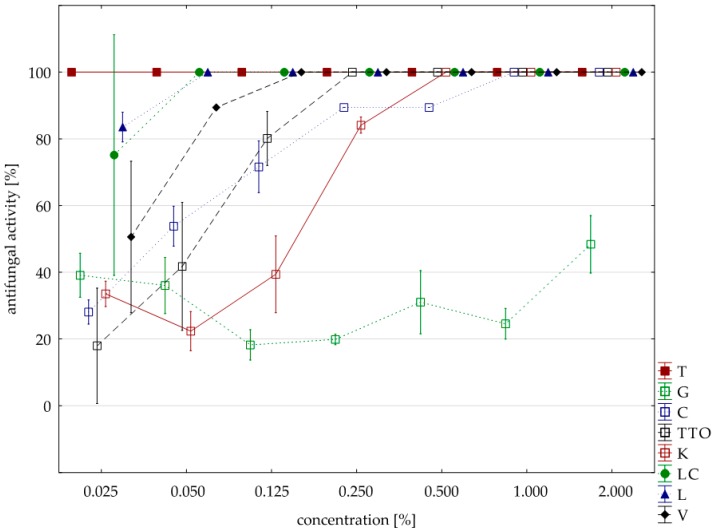
Plot of antifungal activity vs. concentration for *F. graminearum*.

Sensitivity to oils with *F. graminearum* was comparable to *F. culmorum* isolate. Seven oils (except for the grapefruit) showed a 100% fungistatic effect (fungicidal effect). Identical to both isolates were five oils, i.e., thyme, cajeput, lemongrass, *Litsea cubeba*, and verbena. Other effects, depending on the concentration applied, showed two oils, i.e., garlic acted on *F. graminearum* only at a higher concentration (1.000% versus 0.500%) and tea tree. at a lower concentration (0.250% versus 0.500%). In this case, too, grapefruit oil had the weakest effect, and at a concentration of 2.000%, it showed lower fungistatic activity of 48.38% on average (Table 6, Figure 3).

It was hypothesized that differences in fungistatic oils activity on the *F. graminearum* isolate are statistically significant. The result of Kruskal–Wallis test (H (9, n = 231) = 121.67, *p* = 0.00) showed that the hypothesis was true. Differences in fungistatic activity of the eight oils were statistically significant. Moreover, the Kruskal–Wallis test (H (3, n = 88) = 4.96, *p* = 0.17) confirms that there are no statistically significant differences between Funaben T fungistatic activity and thyme, lemongrass and *Litsea cubeba* oils.

**Figure 4:**
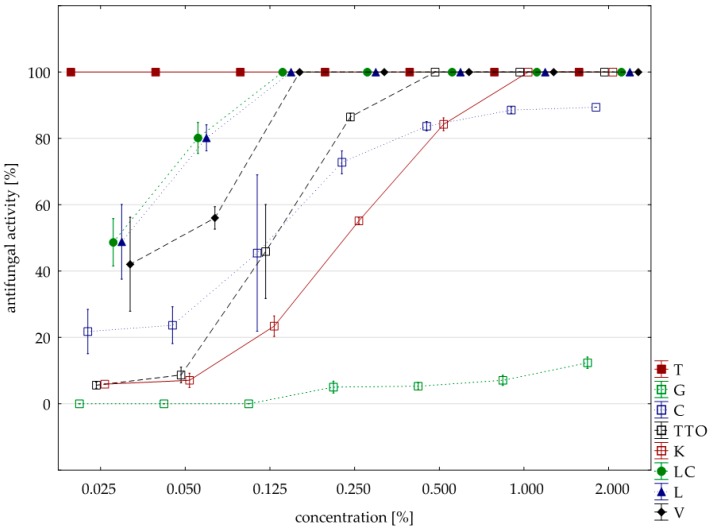
Plot of antifungal activity vs. concentration for *F. avenaceum*.

Significantly lower sensitivity to the tested oils compared to the two previously discussed isolates showed *F. avenaceum*. In this case, six oils (except for the grapefruit and garlic) had a fungicidal activity, but they had to be applied in concentrations higher than the previously discussed *F. culmorum* and *F. graminearum*. Only two oils, thyme and verbena, in concentrations 0.025% and 0.125%, respectively, showed identical fungistatic activity. The remaining four oils (garlic, tea tree, cajeput, lemongrass and *Litsea cubeba*) were twice as concentrated as *F. graminearum* and inhibited the development of *F. avenaceum*. In this case, too, grapefruit oil had the weakest effect; at the highest concentration applied (2.000%), its average fungistatic activity was only 12.35%; garlic oil in the highest concentration (average activity 89.41%) (Table 7, Figure 4). The Kruskal–Wallis tests (H (9, n = 229) = 133.13, *p* = 0.00) indicated that differences in fungistatic activity of the oils in sensitivity of individual isolates with *F. avenaceum* were statistically significant. In addition, the U Mann–Whitney test showed that there are no differences between Funaben T and thyme oil (Z = 0.00, *p* = 1.00).

**Figure 5:**
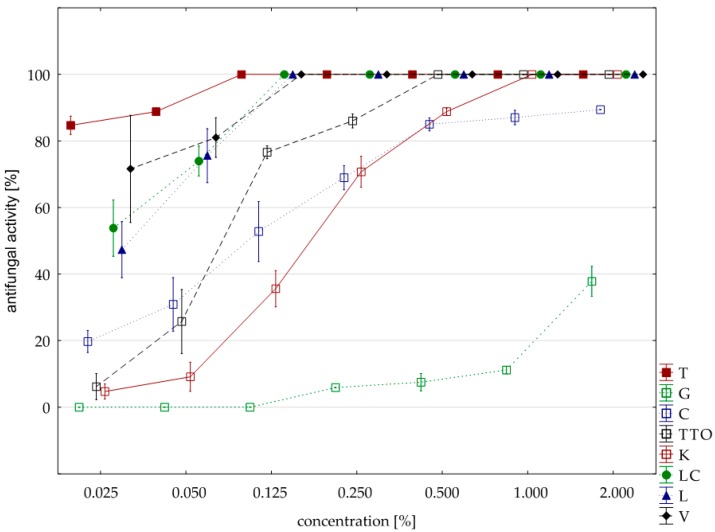
Plot of antifungal activity vs. concentration for *F. oxysporum*.

*F. oxysporum* turned out to be the most resistant of the studied isolates; its resistance was comparable to the one of *F. avenaceum*, although in this case six oils (except for the grapefruit and garlic) showed fungicidal effect. Six oils had the same effect on both isolates, i.e., garlic, tea tree, cajeput, lemongrass, *Litsea cubeba*, and verbena. The isolate of *F. oxysporum* showed exceptionally high resistance to thyme oil, which only in the concentration of 0.125% showed fungicidal activity (on the remaining isolates, it acted in the concentration of 0.025%). Grapefruit oil, however, had a stronger effect than against *F. avenaceum*. The highest concentration (2.000%) had an average fungistatic activity of 37.79%. Garlic oil had the highest concentration with an average activity of 89.41% (Table 8, Figure 5).

The Kruskal–Wallis test ((H (9, n = 231) = 121.92, *p* = 0.00) confirms that differences in fungistatic activity of the eight oils used were statistically significantly. In addition, small differences with thyme oil are visible to *Litsea cubeba* oils, and lemongrass. The Kruskal–Wallis test confirms that there are no statistically significant differences between Funaben T fungistatic activity and thyme, lemongrass, verbena, and *Litsea cubeba* (H (4, n = 115) = 1.89, *p* = 0.76).

### 2.4. Fungistatic Activity of Oils: Combined Analysis of Fusarium Isolates

**Figure 6:**
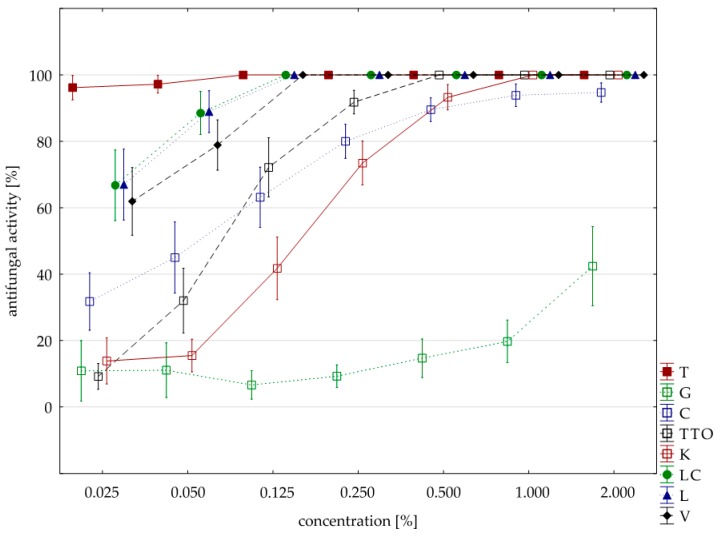
Plot of antifungal activity vs. concentration for four species from genus *Fusarium* (combined analysis).

All tested oils showed fungistatic activity, but their properties were very diverse. The analysis of the fungistatic activity of the oils on the tested isolates showed that the effectiveness of the oils depended on the concentration used. The highest effectiveness (100%), comparable to Funaben T, was recorded for thyme, *Litsea cubeba*, lemongrass, and verbena oils with concentrations from 0.125%. The remaining oils completely inhibited the development of fungi only in higher applied concentrations (Table 9, Figure 6).

**Figure 7:**
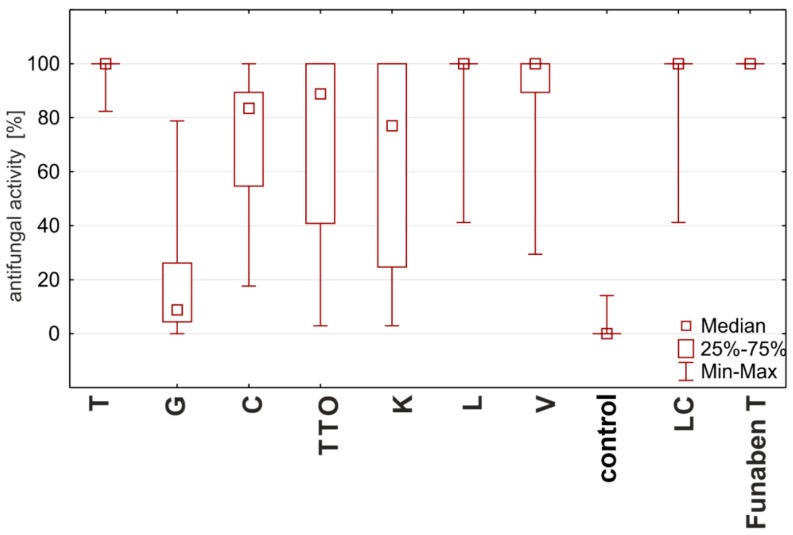
Characteristics of the fungistatic activity of the tested essential oils and Funaben T in relation to isolates of *Fusarium* ssp. [%]: T—thyme; G—grapefruit; C—garlic; TTO—tea tree; K—cajeput; L—lemongrass; V—verbena; LC—*Litsea cubeba*.

Based on the degree of influence of individual oils on the tested *Fusarium* ssp. isolates (expressed as fungistatic activity), three types of interactions can be distinguished. The first type—the strong action—is characterized by oils with concentrations from 0.125%, such as thyme, lemongrass, *Litsea cubeba*, and verbena, showing the highest similarities to the effect of Funaben T (Figure 7). At the same time, the effect of thyme oil seems to be the highest.

The second, lower type of effect—the medium effect—is exhibited by tea tree and cajeput oils. These oils completely inhibited the development of fungi only in higher applied concentrations, i.e., tea tree oil from 0.05% and cajeput oil from 1.0% concentration (Figure 7).

On the other hand, the third type—the weak effect—with the lowest activity is characterized by garlic and grapefruit oils. The effect of these oils on the tested isolates should be considered the weakest as none of the concentrations applied showed 100% effectiveness, but the effect of grapefruit oil was much weaker (Figure 7).

The Kruskal–Wallis test showed that there are statistically significant differences between the fungistatic activity of the oils used for the four isolates tested (H (9, n = 921) = 486.05, *p* = 0.00). Analysis of frame-to-gorchart showed that the greatest similarities to Funaben T performance show thyme, lemongrass, verbena, and *Litsea cubeba*. Statistical analysis showed that only thyme oil does not have significant differences in fungistatic activity compared to Funaben T for all the fungi isolates at issue (Mann–Whitney Test Z = −0.401, *p* = 0.69).

On the basis of the correlation coefficient, a linear relationship between the concentrations of oils and their fungistatic activity in relation to *Fusarium* isolates was found (Table 10). For Funaben T, no correlation coefficient was determined because the fungistatic activity was 100% (showed fungicidal activity) regardless of the concentration used. A relatively low correlation coefficient (0.22–0.35) was found in oils (thyme, *Litsea-cubeba*, lemongrass, and verbena), which were characterized by high effectiveness in the applied concentrations. This means that in practice, it is not advisable to select higher concentrations. On the other hand, the high value of the coefficient correlations for the following oils: cajeput (r = 0.72), grapefruit (r = 0.61), garlic, and tea tree (r = 0.59) suggests that increased oil concentration will significantly affect their fungistatic activity, which practically means that at properly selected concentrations even the ‘weak’ oils can be effective (Table 10).

The presented model of multiple regression showed that the concentration of oils and their chemical composition significantly affect fungistatic activity (Table 11). The concentration of essential oil, monoterpenoids, and sesquiterpenes are directly proportional to antifungal activity. The model describes only 60% of the variability of the antifungal activity (value of R^2^ in Table 12), the remaining 40% variation of dependent variable is not included in the model. However the value of the standard error of estimation is large (Table 12). The model requires detailed research.

## 3. Discussion

An attempt to reduce crop losses caused by pathogenic fungi and at the same time the increasingly evident defects of synthetic biocides lead to a constant search for natural substances limiting the development of fungi. The risk of spreading dangerous pathogens (including *Fusarium*) is increased by the fact that they can gradually become resistant to synthetic disinfectants and pesticides [13], and most of them are very ecotoxic. Danielewicz et al. [17] tested the sensitivity of six *Fusarium* species (*F. avenaceum* KZF-3, *F. culmorum* KZF-5, *F. graminearum* KZF-1, *F. oxysporum* KZF-4, *F. langsethiae* KZF-2, and *F. equiseti* KZF-6) to six fungicides from four chemical groups: azoxystrobin, prochloraz, thiophanate methyl, propiconazole, metconazole, and tebuconazole. These studies showed that the strongest fungicidal effect on all tested *Fusarium* species was found for prochloraz, while thiophanate methyl and azoxystrobin showed only fungistatic activity.

The appearance of individuals (isolates) with reduced sensitivity to fungicides results in the emergence of forms resistant to these preparations. In addition, fungi often have a cross-resistance phenomenon with benzimidazole, dicarboximide and phenylamide fungicides. If a given phytopathogen species becomes resistant to one preparation, at the same time it becomes resistant to the whole group of chemical substances to which this preparation belonged.

The resistance of fungi to chemical compounds determines the specific structure of the cell wall (which protects the cell against external factors) and differences in the set of synthesized and extracellular secreted enzymes [19]. Some chemical fungicides, such as imazalil, prochloraz, and triflumizole, exhibited their antifungal mode by blocking the ergosterol biosynthesis, which can give rise to the disruption of cell structure and function, even to the death of cell [20].

Due to EsO’s wide availability, limitation of the harmful effects of filamentous fungi and biodegradability are increasingly used in practice [21,22]. What is also very important in in vitro experiments are not only their fungicidal activity but also the ability for EsO to degrade mycotoxins [15]. EsO exhibited its antifungal activity by inhibit of hyphal growth, the production and germination of conidia, a change in the morphology of the fungus, damage the cytoplasmic membrane, which lead to the leakage of electrolytes and possibly lipid peroxidation induced by the increase in permeability and the reduction in ergosterol content (major component of the fungal membrane) and accumulation of massive lanosterol as well as an inhibition in wall formation [23,24]. Essential oils also have an inhibitory action on membrane ATPases and cytokine interactions and cell respiration, leading to rapid energy depletion and cell death [25].

Therefore, producing EsO-based biofungicides would help reduce the negative impact of synthetic compounds on food and environmental pollution and reduce risk to biocide-resistant fungi appearance. Therefore, the fungistatic activity of the EsO is extensively tested for *Fusarium* fungi, i.e., *F. avenaceum* [26]; *F. culmorum* [9,27,28,29]; *F. graminearum* [26,28,30]; and *F. oxysporum* [26,27,31,32].

In our research, we used commercial EsO differing in composition and content of chemical compounds. Biological activity of oils with high content of one of three chemical groups was tested, i.e., monoterpenoids (59.02–87.18%), thyme, *Litsea cubeba*, lemongrass, verbena, cajeput, and tea tree oils; monoterpenes (45.64%), grapefruit, organosulphur compounds (100%), and garlic oil.

For the experiment, four *Fusarium* phytopathogenic species, particularly dangerous for crops, were selected—i.e., *F. avenaceum*, *F. culmorum*, *F. graminearum*, and *F. oxysporum*—which were isolated from infected wheat grains produced in the south of Poland. This experiment showed a wide variety of fungistatic activity of EsO against *Fusarium* fungi, which is indicated by both the literature data and the results of the present study.

### 3.1. Composition and Fungistatic Activity of EsO

EsO can contain tens to hundreds of different compounds but three or four main compounds represent more than 60% of the mass and determine the biological activity of essential oils [33]. We can assume that the antimicrobial activity of given EsO may be dependent on only one or two of the main components, making up the oil. In the composition of plant EsO, the substances with the broadest range of biocidal activity were the following: thymol, carvacrol, myrcene, α-terpineol, 1,8-cineole, α-terpinene, terpinen-4-ol, eugenol, linalool, thujone, Δ3-caren, citral, nerol, geraniol, menthone, β-pinene, α-pinene, borneol, sabinene, γ-terpinene, limonene, β-caryophyllene, and p-cymenen [34]. In contrast, carvacrol, thymol and eugenol showed the highest fungicidal (fungistatic) activity [6]. In our experiment, a clear fungicidal activity of oils containing of thymol or citral was confirmed.

There is an increasing number of studies indicating that the activity of EsO may not only depend on one dominant active ingredient but also on the interaction between it and less abundant components. Espina et al., Settani et al., and Białoń et al. [35,36,37] conclude that antimicrobial activity depends not only on the dominant substance but also on the content of monoterpenoids; the higher their content, the stronger the fungicidal effect of the oil. On the other hand, monoterpenes seem to be less antimicrobial active than monoterpenoids [38]. Oils with high content of monoterpenoids (e.g., thyme) were characterized by higher fungicidal activity than grapefruit oil with relatively high content of monoterpenes (limonene) (own studies). Monoterpenes (limonene, α-pinene, β-pinene, δ-3-carene, (+) (−) sabinene, and α-terpinene) showed no or low antimicrobial activity [39]. Some in vitro tests have shown that the use of single compounds as antimicrobials was ineffective [40].

In our study, based on the findings of fungistatic activity of particular low concentrations of oils in relation to tested *Fusarium* isolates, it was shown that the highest similarities to Funaben T were found in oils with high content of monoterpenoids (thyme, *Litsea cubeba*, lemongrass, and verbena); they differed in content and/or type of dominant substances (thymol (45.75%), citral (61.72%), citral (68.94%), and citral (36%) respectively). High content of monoterpenoids (71.66%) was characterized by cajeput oil containing α terpineol (36.57%) as the dominant component, but it showed a fungicidal effect only at the concentration of 1%.

Current research generally showed that the antifungal activity of essential oils against *Fusarium* species depends on the type of EO and its concentration, thus indicating that only some of the tested EOs are able to completely inhibit *Fusarium* growth. However, in the case of TTO, garlic and grapefruit oils, which are less effective than Funaben T, their fungicidal effect increased with increasing concentration, which is also confirmed by the results of studies by Mehani et al. [41].

Thyme oil showed high fungicidal activity in the entire concentration range from 0.025% compared to the three tested isolates (*F. avenaceum*, *F. culmorum*, and *F graminearum*). Only *F. oxysporum* isolate was less sensitive to this oil, used at the lowest concentrations (0.025% and 0.05%). As the results of our experiment show, this species is characterized by the highest resistance among the tested fungi, which is also confirmed by the research of Rai [42]. Kordali et al. [43], however, reported that thymol completely inhibited the growth of mycelium in 17 phytopathogenic fungi, including *F. oxysporum*.

The fungicidal action of the thyme oil tested in our studies may be determined by the ratio of monoterpenoids to monoterpenes (about 2:1) and/or the presence of thymol and limonene in a ratio of 3:1. The fungicidal activity of thyme oil associated with a high thymol content was also demonstrated by Abbaszadeh et al. [44] and Campos-Requena et al. [45]. Whereas Marei et al. [46] indicated promising antifungal activity of thymol (monoterpenoid) together with limonene (monoterpen) [34].

Thyme and clove oils, as well as pure citral, eugenol, and thymol at 500 μL/L, exhibited the highest antimicrobial activity against seven isolates *F. oxysporum* isolates [31].

Pattnaik et.al [47] tested five aromatic essential oil ingredients (cineol, citral, geraniol, linalool, and menthol) for antimicrobial action against 18 bacteria and 12 fungi. It showed their varying efficacy against bacteria and fungi. Against fungi the citral and geraniol oils were the most effective (inhibiting all 12 fungi), followed by linalool (inhibiting 10 fungi), cineole and menthol (each of which inhibited seven fungi) compounds.

EsO containing cis- and trans-citral isomers in significant amounts are known for their fungicidal properties [35,36,48]. Lemongrass oil and citral showed good fungicidal activity against *F. solani* and *F. oxysporum* [49]. Our research showed that lemongrass and *Litsea cubeba* oils had similar effects, in which citral was the dominant substance. In *Listsea cubeba* oil, the ratio of monoterpenoids to monoterpenes, similar to thyme oil, was 2:1, and the presence of citral was accompanied by 3:1 limonene. However, in the case of lemongrass oil, the ratio of monoterpenoids to monoterpenes was 17:1, and the presence of citral was accompanied by linalool in a ratio of about 14:1. This suggests that the fungicidal effect of these oils may be due to the synergy of citral and limonene or citral and linalool.

Reports on the increased antimicrobial activity of EsO as compared to the mixtures of their main single components suggest that components in EsO that are present in low concentrations may also be crucial for the effect of EsO. In this case, synergistic effects can be expected [39,50]. Synergism between aromatic plant components often plays an important role in the effectiveness and reduction of the developing resistance of the pathogen. Therefore, some ingredients, such as carvacrol, γ-terpene, and p-cymene, are more effective when combined [51]. Essential oil of the species *Thymus algeriensis* Boiss. et Reut. (Lamiaceae), which grows wild in Libya, has a stronger biocidal effect than its main components (thymol and carvacrol) used individually, which may indicate synergism in the action of these components [52].

Slightly different conclusions can be drawn from the work of Segvić et al. [53], in which it was shown that thymol had about three times stronger inhibition of pathogen growth than thyme oil. According to these observations, we can speculate that a strong antifungal activity of thyme oil can be attributed to thymol itself or, alternatively, speculate that such a strong fungistatic activity.

In contrast, small amounts of EsO components may also cause antagonistic interactions, which have been observed when comparing the antimicrobial activity of pure carvacrol with the oregano oil, in which carvacrol is the main component. Carvacrol was shown to be 1500 times more effective than crude EsO [40].

In our own research, the least effective in limiting the development of the four tested *Fusarium* species were grapefruit, tea tree, and garlic oils. Sadowska et al. [54] found that grapefruit and tea tree oils in concentrations below 0.2% did not show fungistatic properties towards *F. oxysporum*. However, there are reports of antifungal efficacy in tea tree oil, inhibiting growth of *F. culmorum* [9] and *F. oxysporum* [55]. Grapefruit oil is known for its strong inhibition of growth of other fungi species [56]. Seseni et al. [57] demonstrated a differentiated effect of 10 EsO on *Fusarium* fungi (*F. oxysporum* and *F. circinatum*), whereas the weakest fungistatic properties had citrus oils (mandarin, grapefruit, and orange), and the strongest ones had the oil of clove, thyme and lemongrass.

### 3.2. Additional Comments

Different susceptibility of *Fusarium* to EsO both between individual species and within isolates belonging to the same species and isolated from crops grown in the same habitat conditions [14,58] makes the interpretation of the results difficult. Our own studies showed higher resistance of two isolates: *F. oxysporum* and *F. avenaceum*; the others (*F. culmorum* and *F. graminearum*) reacted to lower concentrations of EsO.

It should also be noted that the chemical composition of EsO from the same plant species and produced by different producers may differ significantly in terms of quality and quantity [4,5,59], which affects their biological activity. That is why there is such a variety of experimental schemes carried out in the field of determining the influence of oils on pathogenic microorganisms.

Regardless of the discussion of some of the results obtained and the differences in opinions of individual researchers, bio-preparations based on EsO are already being produced. Such commercial bio-preparations, e.g., Biosept 33SL (produced by Cintamani, Piaseczno, Poland), containing grapefruit extract, Timorex Gold (produced by Biomor Ltd., Qatzerin, Israel), containing tea tree oil, and Bioczos BR produced by Himal, Łódź, Poland), containing garlic extract. They have a long-lasting inhibitory effect on many species of the *Fusarium*, i.e., *F. avenaceum*, *F. culmorum*, *F. graminearum*, *F. oxysporum*, and *F. poae* [22].

## 4. Materials and Methods

### 4.1. The Research Material

- Four isolates of *Fusarium* (*F. avenaceum* GM2, *F. culmorum* KP17, *F. graminearum* L22, and *F. oxysporum* P6) isolated from infected wheat grains from southern Poland (location see Table 1);
- Commercial EsO of varying chemical composition, i.e.,: thyme (T), *Thymus vulgaris* (produced by MELASAN, Eugendorf, Austria); lemongrass (L), *Cymbopogon citratus* (Lemongrass), *Litsea cubeba* (LC), *Litsea cubeba*, and grapefruit (G), *Citrus paradisi* (produced by TAOASIS GmbH, Berlin, Germany); verbena (V), *Lippia javanica* (produced by Piping Rock Health Products, LLC, Ronkonkoma, NY 11779 USA) garlic (C), *Allium Sativum* (produced by CAELO, Hilden, Germany); tea tree (TTO), *Melaleuca alternifolia* (produced by MEDESIGN IC GmbH Dietramszell—Linden, Germany); cajeput (K), *Melaleuca leucadendron var. cajaputi* (produced by PRIMAVERA LIFE GmbH, Oy-Mittelberg, Germany); at the following concentrations: 0.025; 0.05; 0.125; 0.25; 0.50; 1.0; and 2.0%. The oil colloid solutions were prepared in water with 0.05% Tween 80 (produced by BTL, Poland) and fed into a liquefied PDA medium (Patato Dextrose Agar (BIOCORP, Warszawa, Poland).
- Chemical seed treatment Funaben T (Zakłady Chemiczne “Organika Azot” S.A., Jaworzno, Poland), containing 20% carbendazim and 45% thiocarbamate, applied in concentrations lower, higher and recommended by the manufacturer (0.125, 0.25, and 0.5%). It was a relative control of the effectiveness of EsO.

### 4.2. Procedure for Obtaining Biological Material

Phytopathogenic *Fusarium* species were isolated from infected wheat grains and collected in the south of Poland between 2012 and 2014. The grains were sterilized with 70% ethanol for 20 s and rinsed several times with sterile distilled water. Then, they were dried on sterile tissue paper and placed on Petri dishes with PDA medium with 50 mg of streptomycin dm^−3^ medium. After 24–36 h of incubation at 25 °C, the grown fungal colonies were isolated and transferred to peptone-glucose agar with Bengal rose (produced by BTL, Warszawa, Poland). The collection was then carried out in pure cultures (monosporic cultures) according to the procedure of Tousson and Nelson [60] and stored on PDA slants at 4 °C. The isolated fungi were determined to the species on the basis of macro- and microscopic features according to the studies by Leslie et al. [61] and Watanabe [62]. The species of the dominant *Fusarium* isolates was confirmed by MALDI–TOF. The analysis was performed using MALDI-TOF mass spectrometry with the use of laser desorption/ionization supported with a matrix with time-of-flight analyzer, using a MALDI-TOF mass spectrometer Microflex LT from Bruker Daltonik GmbH, Bremen, Germany). When the procedure of species determination was completed, using taxonomical names of species for particular isolates, their code symbols were removed.

In the study, 4 species of *Fusarium* fungi were used, the most numerous one on wheat grains in south of Poland, for which the identification index value was registered in the range of 2.3–3.00, which guaranteed reliable species determination.

Isolates were stored on PDA slants at 4 °C and transplanted every two months.

### 4.3. Determination of the Quantitative Chemical Composition of Essential Oils

Qualitative and quantitative determination of the chemical composition of EsO was performed using gas chromatography coupled with gas chromatography–mass spectrometry (GC–MS), with HP6890 gas chromatograph coupled with HP 5973A mass spectrometer (Hewlett–Packard, Waldbronn, Germany). Non-polar capillary column HP-5MS (5% diphenyl 95% dimethylpolysiloxane), with a length of 30 m, internal diameter of 0.25 mm, and film thickness of 0.25 μm, was used. Helium was used as the carrier gas. Analyses were carried out in the temperature range 60–280 °C at the heating rate of 10 °C/min^−1^. Further, 1 μL of the tested solutions in dichloromethane was introduced in the ratio 1:50 (*v*/*v*). The type of solvent used did not affect the chemical composition of the tested preparations. The components were identified by comparison of their mass spectra with the spectrometer database of the NIST 11 Library (National Institute of Standards and Technology, Gaithersburg, MD, USA) and by comparison of their retention index calculated against n-alkanes (C9–C20). Each chromatographic analysis was repeated three times. The average value of the relative composition of the essential oil percentage was calculated from the peak areas (Cal). Literature values of Kovats retention indexes (L) based by Babushok et al. [18]. The analysis was repeated three times for each sample [37]. The distribution of oil components was adapted to the studies of Breitmaier [63] and Kohlmunzer [64].

### 4.4. Determination of Fungistatic Activity of Essential Oils

The fungistatic activity of EsO was assessed by using the method of poisoned substrates [65,66]; inoculum was placed on the surface of the oil-modified PDA medium.

The inoculum in the form of 10 mm diameter of media rings overgrown with *Fusarium* mycelium was used in the study. The spore suspension of tested *Fusarium* in a 0.01% sterile solution of Tween 80 (produced by BTL, Warszawa, Poland) was obtained from a 10-day-old culture. The hemocytometer Thoma was used to obtain a spore suspension of 1 × 10^6^ CFU⸱cm^3^. Petri dishes (9 cm diameter) containing 20 cm^3^ PDA medium were inoculated in spore suspension and incubated at 25 °C for 10 days. Then, the discs (of 10 mm diameter) were cut out with a cork borer. Inoculum rings with a diameter of 10 mm overgrown by mycelium were obtained.

Tested EsO were introduced into a PDA medium in the following concentrations: 0.125; 0.25; 0.5, 1.0, and 2.0%. The positive control was the PDA medium with Funaben T (chemical seed mortar) in concentrations of 0.125, 0.25, and 0.5% and the negative control was the culture of the fungus on PDA medium (without oils) enriched with 0.01% Tween 80 with inoculum rings.

The cultures were incubated at 25 °C and every two days until the surface of the medium in the control plates is overgrown, the diameter of developing colonies in two perpendicular directions was measured [9].

The tests were performed in four repetitions (*n* = 4), taking as a repetition one Petri dish from the inoculum in the form of a disc overgrown with pathogen mycelium.

The fungistatic activity of the tested oils was evaluated on the basis of the percentage of inhibition of fungal colony growth calculated from the Abbott formula [67]:

I = (K − C)/K∙100

I—inhibition coefficient—growth stimulation [%]
K—diameter of the fungus colony on the control plate [mm]
C—diameter of the fungus colony on the plate with the given oil [mm].

### 4.5. Statistical Methods

Statistical analysis of the fungistatic activity indices of each of the eight tested EsO at different concentrations was performed. Each experimental variant was repeated four times for four particular species. For each experimental variant, the values of descriptive statistics (mean, median, mode, standard deviation, minimum value, and maximum value) were determined.

The Shapiro–Wilk test was applied to examine the normality of distribution of the fungistatic activity of individual oil. Next, to test if there were any differences between the fungistatic activity of individual oil and the action of Funaben T. Control of each of the mycelium isolates from the non-parametric Kruskal–Wallis test was applied. When the test results were significant, a multiple comparison of mean ranks for all groups (the post-hoc test) was used to discover which pairs of EsO were different from each other. After elimination of the oils that differed from the others, the Kruskal–Wallis test or the Mann–Whitney U test was reused to investigate whether there were still statistically significant differences between other EsO. In order to examine whether the concentration of particular oil affects its fungistatic activity, the correlation coefficients were determined.

Additionally, the linear relationship between the percentage share of a given group of compounds in the EsO and the fungistatic activity of given oil was investigated. For this purpose, the chemical compounds contained in the EsO used were divided into monoterpenes, terpenoids, sesquiterpenes, sesquiterpenoids, Sulphur compounds, and other compounds, and differentiation was made between the main groups of compounds. Next, the Spearman correlation coefficients between the fungistatic activity of given oil and the percentage content of a given group of compounds in that oil were determined. All statistical analyses were performed using STATISTICA, version 13.0 (StatSoft, Inc, Tulsa, OK, USA) at the significance level 0.05.

## 5. Conclusions

All of the tested oils showed fungistatic activity, but their activity varied. The chemical composition of selected commercial oils was diversified. The dominant fraction except for grapefruit and garlic oils were monoterpenoids. The highest effectiveness, fungicidal effect, comparable to Funaben T, was recorded for thyme, *Litsea cubeba*, lemongrass, and verbena oils with concentrations from 0.125%. Their dominant component belonged to the group of monoterpenoids. Higher resistance to low essential oils (EsO) concentrations was characteristic for *F. oxysporum* and *F. avenaceum*, and sensitivity for *F. culmorum* and *F. graminearum*. Fungicidal activity of two monoterpenoids, thymol and citral, dominating in the tested oils, was confirmed.

In view of the excessive use of synthetic pesticides, it seems necessary to carry out systematic monitoring of cereal crops. EsO hide huge potential, which can be used in the reconstruction of allelopathic bonds occurring in nature. Knowledge of these compounds can be a powerful tool in maintaining ecosystems homeostasis.

## Figures and Tables

**Table 1:** Identification results of *Fusarium* species isolated from wheat kernels from the south of Poland. MALDI-TOF: matrix-assisted laser desorption/ionization time of flight mass spectrometer.

No.	Symbol	Place of Isolation	Number of Isolates	Classic Identification	Value of the MALDI-TOF Identification Indicator
1	GM2	Głubczyce	5	*F. avenaceum*	2.49
2	P6	Kietrz	4	*F. oxysporum*	2.38
3	KP17	Kędzierzyn	6	*F. culmorum*	2.61
4	L22	Łosiów	4	*F. graminearum*	2.53

**Table 2:** Chemical composition of the tested essential oils in [%]: T—thyme; L—lemongrass; LC—*Litsea cubeba*; V—verbena; TTO—tea tree; K—cajeput; G—grapefruit.

Compound	RI		Etheric Oils						
	Lit *	Cal *	T	L	LC	V	TTO	K	G
**Monoterpenes**									
tricyclene	923	920	0.17 ± 0.01	0.44 ± 0.08		0	0		
α-thujene	928	928				0.44 ± 0.05	0.83 ± 0.07		
α-pinene	936	933	2.75 ± 0.09	0.49 ± 0.11	2.86 ± 0.16	0	3.42 ± 0.06	5.37 ± 0.01	3.27 ± 0.01
camphene	950	947	1.93 ± 0.07	3.71 ± 0.06	0.58 ± 0.04	0.80 ± 0.02	0		
β-pinene	978	974	0.65 ± 0.02	0	3.95 ± 0.08	1.08 ± 0.05	0.81 ± 0.02	3.93 ± 0.15	
β-myrcene	989	991	2.44 ± 0.03				0.38 ± 0.11	3.01 ± 0.07	5.32 ± 0.01
α-phellandrene	1004	1002	0.87 ± 0.03			0.05 ± 0.02	0.15 ± 0.08		
sabinene (4,10-thujene)	1004	1009				0.27 ± 0.04	0.17 ± 0.03		1.56 ± 0.03
3-carene	1011	1005					17.04 ± 0.15		
α-terpinene	1017	1018	2.32 ± 0.10				10.29 ± 0.09		
p-cymene	1024	1020				3.62 ± 0.03			
limonene	1029	1026	15.15 ± 0.18		20.94 ± 0.13				34.63 ± 0.73
γ-terpinene	1060	1061	8.10 ± 0.07			2.02 ± 0.07		0.37 ± 0.04	0.78 ± 0.01
terpinolene	1087	1087				0.45 ± 0.01	3.87 ± 0.05	0.27 ± 0.02	0.08 ± 0.01
β-patchulene	1457	1455					0.16 ± 0.04		
Sum monoterpenes			34.38	4.64	28.33	8.73	37.12	12.95	45.64
**Monoterpenoids**									
α and β citral (geranial and neral)	-	-		68.94 ± 0.10	61.72 ± 0.43	36.00 ± 0.08			
trifluorolavandulol		1999							2.19 ± 0.07
eucalyptol	1031	1027				13.46 ± 0.17	13.90 ± 0.15	18.50 ± 0.05	
linalool oxide	1065	1064						0,12 ± 0,03	
linalool	1099	1105	8.90 ± 0.18	5.73 ± 0.22	2.58 ± 0.04	8.53 ± 0.01		11.19 ± 0.17	4.83 ± 0.039
1-terpineol	1137	1135	1.19 ± 0.05		1.39 ± 0.06	0.47 ± 0.01		0.87 ± 0.10	
p-menth-3-en-9-ol	1141	1140		0.71 ± 0.02					
camphor	1143	1141				4.62 ± 0.03			
verbenol	1145	1145							0.18 ± 0.014
β-citronellal	1154	1152			1.87 ± 0.13				0.42 ± 0.0.03
borneol	1166	1168	3.07 ± 0.09	2.93 ± 0.07		1.32 ± 0.02			
1-terpinen-4-ol	1177	1181				4.51 ± 0.05	38.24 ± 0.38	4.41 ± 0.35	0.09 ± 0.003
α-terpineol	1190	1197	1.14 ± 0.09		1.02 ± 0.06	18.26 ± 0.150	6.88 ± 0.04	36.57 ± 0.21	1.83 ± 0.048
α-pinene oxide	1197	1195							0.51 ± 0.029
cis-geraniol	1238	1234							0.55 ± 0.046
β citral (neral)	1242	1231							0.92 ± 0.058
trans-geraniol	1255	1252							0.45 ± 0.04
linalyl acetate	1255	1260	0.93 ± 0.06						1.87 ± 0.028
geranial	1270	1269							1.36 ± 0.022
thymol	1290	1298	45.75 ± 0.18						
α-terpinyl acetate	1347								0.23 ± 0.021
nerol acetate	1363	1366		1.68 ± 0.01					
geraniol acetate	1380	1385							2.26 ± 0.045
Sum momoterpenoids			60.98	79.99	68.58	87.18	59.02	71.66	17.69
**Sesquiterpenes**									
α-cubebene	1351	1350						0.52 ± 0.04	0.37 ± 0.004
α-longipinene	1352	1350		0.67 ± 0.08					
ylangene	1370	1370						0.51 ± 0.01	
β-cubebene	1387	1390							0.49 ± 0.028
β-elemene	1388	1387						0.14 ± 0.05	
longifolene	1407	1408				1.12 ± 0.02			
α-gurjunene	1409	1410					0.23 ± 0.02	1.19 ± 0.09	
caryophyllene	1419	1423	4.31 ± 0.02	3.76 ± 0.012	2.45 ± 0.018	0.55 ± 0.06	0.28 ± 0.003	2.60 ± 0.19	0.98 ± 0.061
α-caryophyllene	1420	1408	0.33 ± 0.03	0.45 ± 0.01	0.20 ± 0.004			1.70 ± 0.03	0.14 ± 0.014
β-gurjunene	1431	1430		1.15 ± 0.05			1.23 ± 0.05	0.57 ± 0.03	
(+)aromadendrene	1441	1440					0.94 ± 0.10		
γ-elemene	1449	1445						0.05 ± 0.01	
allo-aromadendrene	1460	1458					0.23 ± 0.03	3.63 ± 0.06	
γ-muurolene	1476	1478					0.12 ± 0.06		
germacene D	1481	1496						0	0.18 ± 0.01
(+)-valencene	1491	1499		0					0.14 ± 0.09
β-selinene	1493	1490						1.62 ± 0.03	
γ-cadinene	1513	1517		4.83 ± 0.10					
σ-cadinene	1523	1526					0.83 ± 0.04		0.48 ± 0.009
cadinene	1533	1530						0.37 ± 0.05	
Sum sesquiterpenes			4.64	10.86	2.65	1.67	3.86	12.90	2.83
**Sesquiterpenoids**									
trans-nerolidol	1524	1522							0.02 ± 0.006
elemol	1536	1540							0.05 ± 0.01
caryophyllene oxide	1581	1572		1.14 ± 0.03	0.44 ± 0.05			0.42 ± 0.08	0.23 ± 0.003
guaiol	1589	1590						0.55 ± 0.05	
eudesmol	1616	1611						1.52 ± 0.03	
farnesol	1722	1718							0.05 ± 0.011
nootkatone	1813	1818							1.37 ± 0.069
farnesyl acetate	1818	1820							0.03 ± 0.002
Sum sesquiterpenoids				1.14	0.44			2.49	1.75
Sum other chemical compounds				3.37					26.81

Lit *—Literature values of Kovats retention indexes [18]. Cal *—The average value of the relative composition of the essential oil percentage was calculated from the peak areas.

**Table 3:** Main chemical groups in thyme (T), lemongrass (L), *Litsea cubeba* (LC), verbena (V), tea tree (TTO), cajeput (K), garlic (C), grapefruit (G) oils.

Main Groups of Compounds	Etheric Oils							
	T	L	LC	V	TTO	K	C	G
monoterpenes	34.38	4.64	28.33	8.73	37.12	12.95	0.00	45.64
monoterpenoids	60.98	79.99	68.58	87.17	59.02	71.66	0.00	17.69
sesquiterpenes	4.64	10.86	2.65	1.67	3.86	12.9	0.00	2.83
sesquiterpenoids	0.00	1.14	0.44	0.00	0.00	2.49	0.00	1.75
sulfur-organic compounds	0.00	0.00	0.00	0.00	0.00	0.00	100.00	0.00
other chemical compounds	0.00	3.37	0.00	2.42	0.00	0.00	0.00	32.09

**Table 4:** Main groups of terpenes: thyme (T), lemongrass (L), *Litsea cubeba* (LC), verbena (V), tea tree (TTO), cajeput (K), and grapefruit (G) oils.

Detailed Division into Groups of Compounds	T	L	LC	V	TTO	K	G
aliphatic monoterpenes	0	0	0	0	0.38	3.01	5.32
monocyclic monoterpenes	26.44	0	20.94	6.14	14.31	0.64	35.49
bi- and tricyclic monoterpenes	5.5	4.64	7.39	2.59	22.43	9.3	4.83
aliphatic monoterpenoids	9.83	76.35	66.17	44.53	0	11.19	14.85
monocyclic monoterpenoids	48.08	0.71	2.41	23.24	45.12	41.85	2.15
bi- and tricyclic monoterpenoids	3.07	2.93	0	19.40	13.90	18.62	0.69
aliphatic sesquiterpenes	0	0	0	0	0	0	0.05
monocyclic sesquiterpenes	4.64	4.21	2.65	0.55	0.28	4.49	1.30
bi- and tricyclic sesquiterpenes	0	6.65	0	1.12	3.58	8.41	1.48
aliphatic sesquiterpenoids	0	0	0	0	0	0	0.10
monocyclic sesquiterpenoids	0	0	0	0	0	0	0.05
bi- and tricyclic sesquiterpenoids	0	1.14	0.44	0	0	2.49	1.60

**Table 5:** Maximum fungistatic activity of the analyzed oils at minimum concentration of the isolate of *F. culmorum*.

Oil	Concentration	N	Mean	Median	Min	Max	SD
thyme	0.025	4.00	100.00	100.00	100.00	100.00	0.00
grapefruit	2.000	4.00	71.03	73.82	57.65	78.82	9.60
garlic	0.500	4.00	100.00	100.00	100.00	100.00	0.00
tea tree	0.500	4.00	100.00	100.00	100.00	100.00	0.00
cajeput	0.500	4.00	100.00	100.00	100.00	100.00	0.00
*Litsea cubeba*	0.050	4.00	100.00	100.00	100.00	100.00	0.00
lemongrass	0.050	4.00	100.00	100.00	100.00	100.00	0.00
verbena	0.125	4.00	100.00	100.00	100.00	100.00	0.00
control		4.00	0.00	0.00	0.00	0.00	0.00
Funaben T	0.125	1.00	100.00	100.00	100.00	100.00	0.00

**Table 6:** Maximum fungistatic activity of the analyzed oils at minimum concentration for the isolate of *F. graminearum*.

Oil	Concentration	N	Mean	Median	Min	Max	SD
thyme	0.025	4.00	100.00	100.00	100.00	100.00	0.00
grapefruit	2.000	4.00	48.38	47.35	43.53	55.29	5.40
garlic	1.000	4.00	100.00	100.00	100.00	100.00	0.00
tea tree	0.250	4.00	100.00	100.00	100.00	100.00	0.00
cajeput	0.500	3.00	100.00	100.00	100.00	100.00	0.00
*Litsea cubeba*	0.050	4.00	100.00	100.00	100.00	100.00	0.00
lemongrass	0.050	4.00	100.00	100.00	100.00	100.00	0.00
verbena	0.125	4.00	100.00	100.00	100.00	100.00	0.00
control		4.00	0.00	0.00	0.00	0.00	0.00
Funaben T	0.125	1.00	100.00	100.00	100.00	100.00	0.00

**Table 7:** Maximum fungistatic activity of the analyzed oils at minimum concentration for the isolate of *F. avenaceum*.

Oil	Concentration	N	Mean	Median	Min	Max	SD
thyme	0.025	4.00	100.00	100.00	100.00	100.00	0.00
grapefruit	2.000	4.00	12.35	12.35	11.18	13.53	1.07
garlic	2.000	4.00	89.41	89.41	89.41	89.41	0.00
tea tree	0.500	4.00	100.00	100.00	100.00	100.00	0.00
cajeput	1.000	4.00	100.00	100.00	100.00	100.00	0.00
*Litsea cubeba*	0.125	4.00	100.00	100.00	100.00	100.00	0.00
lemongrass	0.125	4.00	100.00	100.00	100.00	100.00	0.00
verbena	0.125	4.00	100.00	100.00	100.00	100.00	0.00
control		4.00	0.00	0.00	0.00	0.00	0.00
Funaben T	0.125	1.00	100.00	100.00	100.00	100.00	0.00

**Table 8:** Maximum fungistatic activity of the analyzed oils at minimum concentration of the isolate of *F. oxyporum*.

Oil	Concentration	N	Mean	Median	Min	Max	SD
thyme	0.125	4.00	100.00	100.00	100.00	100.00	0.00
grapefruit	2.000	4.00	37.79	37.06	35.29	41.77	2.82
garlic	2.000	4.00	89.41	89.41	89.41	89.41	0.00
tea tree	0.500	4.00	100.00	100.00	100.00	100.00	0.00
cajeput	1.000	4.00	100.00	100.00	100.00	100.00	0.00
*Litsea cubeba*	0.125	4.00	100.00	100.00	100.00	100.00	0.00
lemongrass	0.125	4.00	100.00	100.00	100.00	100.00	0.00
verbena	0.125	4.00	100.00	100.00	100.00	100.00	0.00
control		4.00	3.53	0.00	0.00	14.12	7.06
Funaben T	0.125	1.00	100.00	100.00	100.00	100.00	0.00

**Table 9:** Descriptive statistics on the assessment of fungistatic activity of essential oils at the tested oils concentrations in relation to *Fusarium* isolates (combined analysis).

Oil	Concentration	N	Mean	Median	Min	Max	SD
thyme	0.125	16.00	100.00	100.00	100.00	100.00	0.00
grapefruit	2.000	16.00	42.39	42.65	11.18	78.82	22.37
garlic	2.000	16.00	94.71	94.71	89.41	100.00	5.47
tea tree	0.500	16.00	100.00	100.00	100.00	100.00	0.00
cajeput	1.000	16.00	100.00	100.00	100.00	100.00	0.00
*Litsea cubeba*	0.125	16.00	100.00	100.00	100.00	100.00	0.00
lemongrass	0.125	16.00	100.00	100.00	100.00	100.00	0.00
verbena	0.125	16.00	100.00	100.00	100.00	100.00	0.00
control		16.00	0.88	0.00	0.00	14.12	3.53
Funaben T	0.125	4.00	100.00	100.00	100.00	100.00	0.00

**Table 10:** Values of correlation coefficients between the oil concentration and its fungistatic activity in relation to the four tested *Fusarium* isolates.

Oil	Assessment of Fungistatic Activity [%]
thyme	0.22
grapefruit	0.61
garlic	0.64
tea tree	0.59
cayeput	0.72
*Litsea cubeba*	0.35
lemongrass	0.35
verbena	0.33

**Table 11:** Result of multiple regression.

	b *	SE of b *	b	SE of b	t (887)	*p* Value
Intercept			61.601	2.158	28.539	0.000 *
concentration	0.336	0.021	1740.112	109.352	15.913	0.000 *
monoterpenes	−0.107	0.026	−0.233	0.056	−4.119	0.000 *
monoterpenoids	0.257	0.029	0.309	0.035	8.778	0.000 *
sesquiterpenes	0.196	0.053	1.595	0.427	3.736	0.000 *
sesquiterpenoids	−0.467	0.053	−17.827	2.030	−8.783	0.000 *
other chemical compounds	−0.219	0.040	−0.724	0.132	−5.481	0.000 *

Notation: b—regression coefficient; b *—standardized coefficient; SE standard error; t—t-Student test value; *—the result statistically significant.

**Table 12:** Statistic of multiple regression.

Statistic	Value
R	0.777
R^2^	0.604
Adjusted R^2^	0.601
F (6887)	225.15
*p*	0.000 *
Std. Error of estimate	21.775

Notation: R—multiple correlation coefficient; R^2^—coefficient of determination; F—ANOVA of multiple regression value; *—the result statistically significant.
